# Complete mitochondrial genome of *Zeuzera multistrigata* Moore, 1881 (Lepidoptera: Cossidae)

**DOI:** 10.1080/23802359.2017.1361364

**Published:** 2017-08-02

**Authors:** Iksoo Kim, Su Yeon Jeong, Min Jee Kim, Sei-Woong Choi

**Affiliations:** a Department of Applied Biology College of Agriculture & Life Sciences, Chonnam National University, Gwangju, Republic of Korea;; b Department of Environmental Education, Mokpo National University, Muan, Republic of Korea

**Keywords:** Mitochondrial genome, *zeuzera multistrigata*, Cossidae

## Abstract

We sequenced the complete mitochondrial genome (mitogenome) of *Zeuzera multistrigata* Moore, 1881 (Lepidoptera: Cossidae), which is an economically damaging pest to a diverse range of *Casuarina* trees. The 15,260-bp-long mitogenome of this species consists of a typical set of genes, with an arrangement typical of ditrysian Lepidoptera. Of the 13 protein-coding genes (PCGs), 12 have a typical ATN start codon, whereas the *COI* gene begins with CGA. Ten of the 13 PCGs end with TAA, whereas *COI*, *COII*, and *ND5* ended with an incomplete termination codon, T. Phylogenetic analysis using 13 PCGs of available superfamilies in Apoditrysia indicated that *Z. multistrigata*, belonging to the superfamily Cossoidea, formed a sister group with the confamilial species *Eogystia hippophaecolus*; however, the nodal support for this group was low (46%). As more mitogenomes from ancient groups of Lepidoptera become available, further robust phylogenetic analysis and inference will be possible.

*Zeuzera multistrigata,* a moth belonging to the Cossidae family, is found in several Asian countries including Korea (Shin 2001). Adults, which have a wingspan of 40–60 mm, are found from July to August every two years (Shin 2001). The larvae are borers of a diverse range of *Casuarina* trees (Shin 2001).

In this study, the complete mitochondrial genome (mitogenome) of *Z. multistrigata* was sequenced to determine the mitogenomic characteristics of this species and its phylogenetic position within the Lepidoptera. An adult *Z. multistrigata* was captured at Mongtan-myeon, in Jeollanamdo Province, South Korea, in 7 August 2012 (34°55′00″N, 126°27′18″E). This voucher specimen was deposited in Chonnam National University, Gwangju, Korea, under the accession no. CNU5711. Using the total DNA from this specimen as a template, three long-overlapping fragments (*COI*-*ND4* for LF1, *ND5*-*lrRNA* for LF2, and *lrRNA*-*COI* for LF3) were amplified using three sets of Lepidoptera-specific primers adapted from Kim et al. (2014). These LFs were used as templates for 26 short fragments.

The *Z. multistrigata* mitogenome is 15,260 bp in length and includes typical sets of genes and a major non-coding 374-bp A + T-rich region (GenBank accession no. MF491642). The mitogenome size is slightly smaller than that of the available confamilial species *Eogystia hippophaecolus* (Gong et al. 2014), but is well within the range found in apoditrysian Lepidoptera (Timmermans et al. 2014; Liu et al. 2016, 2017). The gene arrangement of the *Z. multistrigata* mitogenome is identical to that of the ditrysian Lepidoptera that have the order *trnM*-*trnI*-*trnQ* in the A + T-rich region and *ND2* junction (Kim et al. 2014). The AT content among genes and regions varies considerably in the *Z. multistrigata* mitogenome (from 93.9% in the A + T-rich region to 84.7% in rRNAs). Twelve *Z. multistrigata* PCGs start with a typical ATN codon, whereas *COI* starts with the atypical sequence CGA, as found in the majority of lepidopteran species (Kim et al. 2014; Park et al. 2016).

We downloaded available mitogenome sequences of 10 apoditrysian species (seven superfamilies) from GenBank along with that of one species of Tineoidea in Ditrysia for use as an outgroup, and 13 PCGs were utilized for phylogenetic analysis. The Maximum-likelihood (ML) method was performed using the GTR + GAMMA + I model and using RAxML-HPC2 in XSEDE ver. 8.0.24 (Stamatakis 2014), which is implemented in the CIPRES Portal ver. 3.1 (Miller et al. 2010). *Zeuzera multistrigata*, belonging to the superfamily Cossoidea, formed a sister group with the confamilial species *E. hippophaecolus*, and these two species of Cossoidea formed a sister group to Urodoidea (Figure 1). With exception of the sister relationships between two species of Zygaenoidea (100%) and between two species of Totricoidea (100%), the nodal supports for most groups, including the sister relationships between the two species of Cossoidea (46%) and between Cossoidea and Urodoidea (17%), were low. In most cases, these results contribute little to the recent progress made in lepidopteran phylogeny (e.g. Bazinet et al. 2013). One possible reason for such results could be the lack of taxonomic diversity. Thus, more mitogenomes from ancient lepidopteran groups could be essential for further robust phylogenetic analysis and inference.

**Figure 1.**
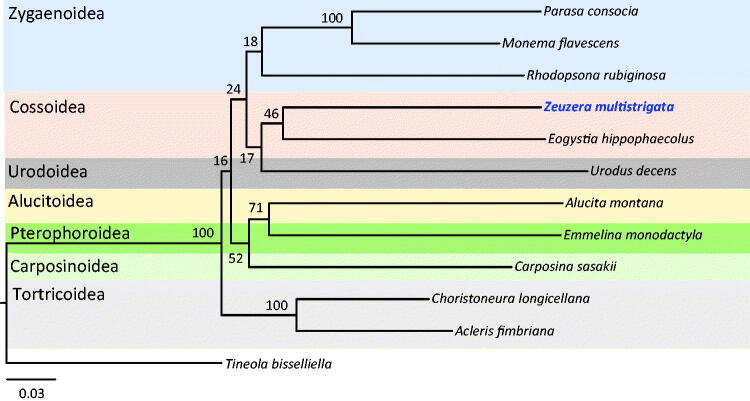
Phylogeny of Apoditrysia, including *Z. multistrigata* belonging to Cossoidea, derived using the Maximum-likelihood (ML) method based on 13 concatenated PCGs. The numbers at each node specify bootstrap percentages of 1000 pseudoreplicates. The scale bar indicates the number of substitutions per site. *Tineola bisselliella* belonging to the superfamily Tineoidea in Ditrysia was utilized as an outgroup. GenBank accession numbers are as follows: *Eogystia hippophaecolus*, KC831443; *Monema flavescens*, KU946971; *Parasa consocia*, KX108765; *Rhodopsona rubiginosa*, KM244668; *Choristoneura longicellana*, HQ452340; *Acleris fimbriana*, HQ662522; *Urodus decens*, KJ508062; *Emmelina monodactyla*, KJ508063; *Alucita montana*, KJ508059; *Carposina sasakii*, HQ840719; and *Tineola bisselliella*, KJ508045.

## References

[CIT0001] Bazinet AL, Cummings MP, Mitter KT, Mitter CW. 2013 Can RNA-Seq resolve the rapid radiation of advanced moths and butterflies (Hexapoda: Lepidoptera: Apoditrysia)? An exploratory study. PLoS One. 8:e82615. 24324810 10.1371/journal.pone.0082615 PMC3853519

[CIT0002] Gong Y-J, Wu Q-L, Wei S-J. 2014 The first complete mitogenome for the superfamily Cossoidea of Lepidoptera: the seabuckthorn carpenter moth *Eogystia hippophaecolus*. Mitochondrial DNA. 25:288–289. 23841606 10.3109/19401736.2013.792071

[CIT0003] Kim MJ, Wang AR, Park JS, Kim I. 2014 Complete mitochondrial genomes of five skippers (Lepidoptera: Hesperiidae) and phylogenetic reconstruction of Lepidoptera. Gene. 549:97–112. 25058696 10.1016/j.gene.2014.07.052

[CIT0004] Liu Q-N, Xin Z-Z, Bian D-D, Chai X-Y, Zhou C-L, Tang B-P. 2016 The first complete mitochondrial genome for the subfamily Limacodidae and implications for the higher phylogeny of Lepidoptera. Sci Rep. 6:35878. 27767191 10.1038/srep35878 PMC5073316

[CIT0005] Liu Q-N, Xin Z-Z, Zhu XY, Bian D-D, Chai X-Y, Zhou X-M, Zhou C-L, Tang B-P. 2017 A transfer RNA gene rearrangement in the lepidopteran mitochondrial genome. Biochem Biophys Res Commun. 489:1498–1154. 10.1016/j.bbrc.2017.05.115 28546004

[CIT0006] Miller MA, Pfeiffer W, Schwartz T. 2010 Creating the CIPRES Science Gateway for inference of large phylogenetic trees. In: Proceedings of the 9th Gateway Computing Environments Workshop (GCE), New Orleans. p. 1–8.

[CIT0007] Park JS, Kim MJ, Jeong SY, Kim SS, Kim I. 2016 Complete mitochondrial genomes of two gelechioids, *Mesophleps albilinella* and *Dichomeris ustalella* (Lepidoptera: Gelechiidae), with a description of gene rearrangement in Lepidoptera. Curr Genet. 62:809–826. 26952721 10.1007/s00294-016-0585-3

[CIT0008] Shin Y-H. 2001 Coloured Illustrations of The Moths of Korea. Seoul: Academybook Publishing Co, Ltd 113p.

[CIT0009] Stamatakis A. 2014 RAxML version 8: a tool for phylogenetic analysis and post-analysis of large phylogenies. Bioinformatics. 30:1312–1313. 24451623 10.1093/bioinformatics/btu033 PMC3998144

[CIT0010] Timmermans MJ, Lees DC, Simonsen TJ. 2014 Towards a mitogenomic phylogeny of Lepidoptera. Mol Phylogenet Evol. 79:169–178. 24910155 10.1016/j.ympev.2014.05.031

